# Effectiveness of Physical Therapy in Orthognathic Surgery Patients: A Systematic Review of Randomized Controlled Trials

**DOI:** 10.3390/jfmk8010017

**Published:** 2023-01-30

**Authors:** Gonzalo Navarro-Fernández, Alfonso Gil-Martínez, Marta Carlota Diaz-Saez, Ignacio Elizagaray-Garcia, Paloma Qinling Pili-Mayayo, Julian Esteban Ocampo-Vargas, Hector Beltran-Alacreu

**Affiliations:** 1 Escuela Internacional de Doctorado, Department of Physical Therapy, Occupational Therapy, Rehabilitation and Physical Medicine, Universidad Rey Juan Carlos, 28922 Alcorcón, Spain; 2 CranioSPain Research Group, Centro Superior de Estudios Universitarios La Salle, Universidad Autónoma de Madrid, 28049 Madrid, Spain; 3 Department of Physiotherapy, Centro Superior de Estudios Universitarios La Salle, Universidad Autónoma de Madrid, 28023 Madrid, Spain; 4 Unit of Physiotherapy, Hospital La Paz-Carlos III, Institute for Health Research IdiPAZ, 28046 Madrid, Spain; 5 Motion in Brains Research Group, Centro Superior de Estudios Universitarios La Salle, Universidad Autónoma de Madrid, 28023 Madrid, Spain; 6 Toledo Physiotherapy Research Group (GIFTO), Faculty of Physical Therapy and Nursing, Universidad de Castilla-La Mancha, 45004 Toledo, Spain

**Keywords:** orthognathic surgery, exercise, rehabilitation, physiotherapy, pain, jaw

## Abstract

Orthognathic surgery (OS) can present many complications that affect patients’ rehabilitation. However, there have been no systematic reviews that assessed the effectiveness of physiotherapy interventions in the postsurgical rehabilitation of OS patients. The aim of this systematic review was to analyze the effectiveness of physiotherapy after OS. The inclusion criteria were randomized clinical trials (RCTs) of patients who underwent OS and who received therapeutic interventions that included any physiotherapy modality. Temporomandibular joint disorders were excluded. After the filtering process, five RCTs were selected from the 1152 initially obtained (two had acceptable methodological quality; three had insufficient methodological quality). The results obtained showed that the effects of the physiotherapy interventions studied in this systematic review on the variables of range of motion, pain, edema and masticatory muscle strength were limited. Only laser therapy and LED showed a moderate level of evidence in the postoperative neurosensory rehabilitation of the inferior alveolar nerve compared with a placebo LED intervention.

## 1. Introduction

Orthognathic surgery is a type of surgical intervention indicated for correcting moderate to severe dentofacial deformities and occlusion problems, and its objective is an appropriate facial balance and proportion, as well as correct functionality [1].

It has been estimated that orthognathic surgery is indicated for functional abnormalities in 52% of cases and for aesthetic reasons in 27% of cases [2]. Typically, the functional abnormalities that indicate the need for orthognathic surgery are due to morphological problems of the maxillary and/or mandibular bone that jeopardize oral function and occlusion [3]. In their 2018 meta-analysis, Alhammadi et al. observed that patients with permanent dentition had a 74.7% prevalence of class I occlusion, 19.56% of class II and 5.93% of class III [4]. In their 2019 study, Asiri et al. observed that in a sample of more than 8000 adult participants, 32% had at least one relevant clinical measure of occlusal problems, approximately 14% showed severe morphological abnormalities, 4.2% showed an excessive overjet (anteroposterior overlapping distance between the maxillary and mandibular incisors [5]) and 1.3% had an excessive overbite (vertical overlapping distance between the maxillary and mandibular incisors [5]) [6]. Other patients who require orthognathic surgery are those who experience maxillofacial fractures. In fact, it has been estimated that in 2017 alone there were more than 7.5 million new cases of maxillofacial fractures worldwide, which resulted in almost 120,000 lost years due to disability [7].

Although there are conservative methods to correct occlusal abnormalities, such as orthodontic treatment, many patients undergo orthognathic surgery to improve their functionality, aesthetics and occlusion [2]. The most common orthognathic surgery approaches are Le Fort 1 osteotomy, sagittal osteotomy of the mandibular branch and genioplasty [1]. A number of complications can appear as a result of the surgical process, such as sensory disorders due to impairment of the trigeminal nerve or facial nerve [1], movement abnormalities [8], pain [9] and especially edema in the face and neck [10], affecting functionality of patients who undergo orthognathic surgery, with a highly variable incidence: 17.8% of patients who undergo this surgery experience pain up to 1 year after the surgery [9] and almost 60% have sensitivity impairment up to 6 months after the surgery [11]. Some authors have, therefore, investigated various therapeutic approaches to reduce the onset of these complications and their impact on patients’ lives, including administration of corticoids [12], cryotherapy [13], manual lymphatic drainage [14] and low-intensity laser [15].

A few published studies have addressed the effectiveness of physiotherapy interventions for postorthognathic surgery patients. Some of the interventions used in these studies aimed to reduce patients’ pain intensity (such as the transcutaneal electrical nerve stimulation [16]) or to improve mandibular range of motion (such as therapeutic exercises [17]). In general, the results of these studies show that the physiotherapy interventions favorably influence the patients’ postsurgical rehabilitation, although not always to a greater degree than in the control group [16,17]. To our knowledge, there have been no systematic reviews to date that assessed the effectiveness of physiotherapy interventions in the postsurgical rehabilitation of patients who have undergone orthognathic surgery.

Therefore, the main objective of this systematic review of randomized clinical trials (RCTs) was to analyze the effectiveness of physiotherapy interventions in the postsurgical mouth opening range of motion (ROM) of patients who have undergone orthognathic surgery. The secondary objective was to analyze the effect of physiotherapy on other postsurgical functional variables, such as neurosensory disorder, myoelectric activity, pain, bite strength and edema, in patients who have undergone orthognathic surgery.

## 2. Materials and Methods

The systematic review was conducted according to the standards of the PRISMA declaration (Preferred Reporting Items for Systematic Reviews and Meta-Analyses) [18]. The protocol of this systematic review was registered in the International Prospective Register of Systematic Reviews (PROSPERO) database (code CRD42021254655).

### 2.1. Inclusion Criteria

The methodological characteristics of the studies of interest for the present review comprised five relevant aspects: (P) population, (I) intervention, (C) comparison, (O) outcome measures and (S) study design. The studies’ population (P) needed to be patients older than 18 years who underwent orthognathic surgery (men and women). The studies’ intervention (I) needed to be any physiotherapy intervention compared (C) with another intervention, placebo or control group. In terms of outcome variables (O), the studies must have assessed at least one of the following variables: maximum mouth opening, sensitivity disorder, myoelectric activity of the masticatory muscles, pain, bite strength or inflammation. Lastly, in terms of study design (S), all articles must have had RCTs. We excluded those studies that conducted surgery on the temporomandibular joint (meniscectomy, arthroscopy, etc.) or oral surgery (e.g., surgery for impaction of the third molar), as well as those studies that included patients with concomitant systemic or neurological conditions.

### 2.2. Search Strategy

A search of RCTs was conducted using the databases MEDLINE, EMBASE, WOS, CINAHL and Google Scholar, with no language limitation. Grey literature sources were also consulted, including OpenGrey and Teseo, to reduce publication biases (no relevant results were obtained). This search phase ended on 25 April 2021.

The following search strategy was employed for each of the listed databases: surgery AND (jaw OR mandibular) AND (rehabilitation OR physiotherapy OR exercise). To cover the largest number of original studies possible, this strategy was combined with the following free terms and descriptors: “Orthognathic surgery”, “physical therapy”, “rehabilitation”, “physiotherapy”, “exercise”, “mandibular OR jaw”, “pain” and “quality of life”.

### 2.3. Selection Criteria and Data Extraction

The data analysis was performed by 2 independent evaluators (JEOV, PQPM) who, after eliminating duplicated RCTs, assessed in the first filtration phase whether the studies answered the question and the objective of this review. This first analysis was performed according to the information obtained from each study’s title, abstract and keywords. When the information was not entirely clear or concise, the study’s complete text was reviewed (when in doubt, the study was always passed on to the next filtration phase). In the second part of the analysis, with the reading of the articles’ complete text, the evaluators checked that all the articles met the inclusion criteria of this systematic review. Disagreements between the reviewers were resolved by a third experienced evaluator (AGM), who operated independently.

### 2.4. Methodological Quality Assessment

The assessment of the studies’ quality was performed using the PEDro scale translated into Spanish [19]. The PEDro scale evaluates the criteria listed in Table 1. These criteria were scored with 1 point if they were met and 0 points if they were not and had to be evaluated for each corresponding RCT. The first item had to have a score of 1 for the study to be accepted, and this item was excluded from the final count (it was used only as a representation of the items of the scale of origin, Delphi). The scores ranged from 0 to 10 points. Based on the recommendations of Cochrane Back and Neck Group, the methodological quality was considered acceptable when the study achieved a minimum score of 6 (more than 50% of the total score of the PEDro scale) [20].

The quality of the articles was evaluated by two independent reviewers using the same methodology (PEDro scale). To determine the correlation between the evaluators, we used the kappa coefficient (κ), considering κ > 0.7 as indicating high agreement between the two evaluators, 0.5–0.7 as indicating moderate agreement and <0.5 as indicating low agreement. The statistical software SPSS v. 25.0 (IBM Inc. Chicago, IL USA) was employed to calculate κ. The differences in the results between the reviewers were resolved by the intervention of a third independent evaluator.

### 2.5. Risk of Bias Assessment

For the risk of bias assessment, two evaluators used the Cochrane Risk of Bias (RoB) tool, which assesses the following types of biases: selection, implementation, detection, wear and notification, among others. Each of the evaluated items were classified as high risk of bias, low risk of bias or undetermined [24].

### 2.6. Qualitative Analysis

The qualitative analysis employed in this review is based on the classification of results according to scientific evidence levels [25]. The evidence was divided into five levels, according to the studies’ results and methodological quality:

(1) Strong evidence: represents results from multiple RCTs with acceptable methodological quality.
(2) Moderate evidence: represents results from multiple RCTs with low methodological quality, controlled clinical trials or high-quality RCTs.
(3) Limited evidence: represents results from an RCT or low-quality controlled clinical trial.
(4) Conflicting evidence: represents conflicting results from an RCT or controlled clinical trials.
(5) No evidence: there are no RCTs or controlled clinical trials.

## 3. Results

**Figure 1:**
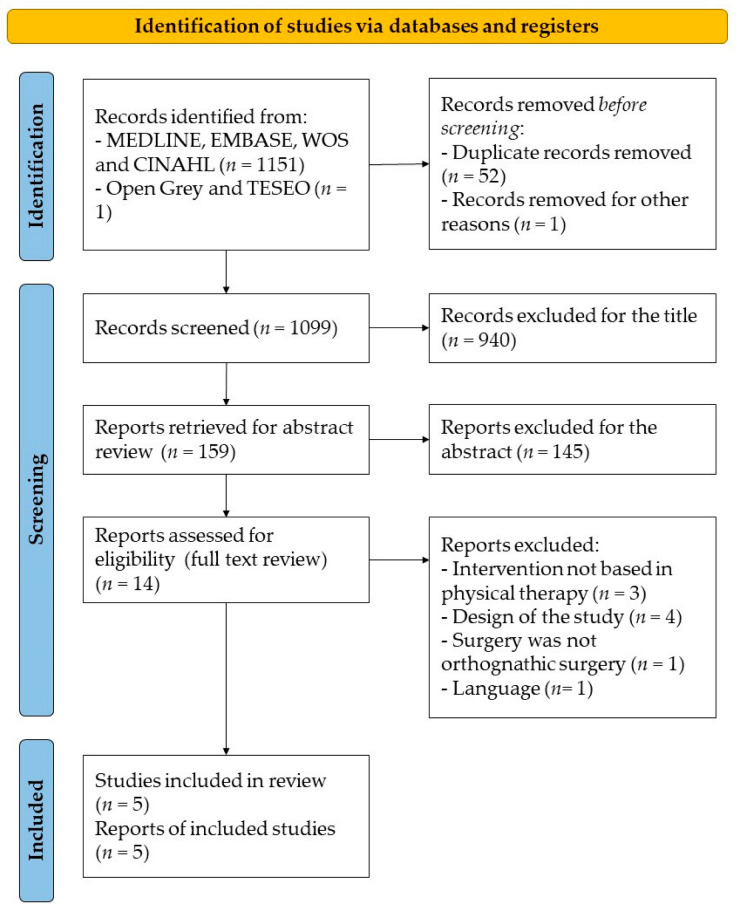
Flow diagram.

Of the 1152 initially identified studies, only 13 were selected during the preanalysis phase. After an exhaustive review of the selected articles, only five met the inclusion criteria of the present systematic review [16,17,21,22,23] (Figure 1). In the five included RCTs, physiotherapy was performed in one of their modalities. Table 2 describes the studies’ epidemiological characteristics, the most relevant results and the authors’ conclusions for each RCT.

### 3.1. Characteristics of the Included Studies

#### 3.1.1. Size and Characteristics of the Sample

All the studies were conducted on populations that underwent orthognathic surgery for various causes, including sagittal osteotomy to correct malocclusion problems [22], surgery with intermaxillary fixation for fractures [16], bimaxillary surgery for cleft palate [23] and bilateral osteotomy of the mandibular branch to correct malocclusion problems [17,21]. All the selected RCTs reported losses and attrition of their participants during the intervention and analysis process. Additionally, two of the RCTs [16,22] reported that they performed the analysis by intent to treat. In total, 155 participants were included (84 women, 54%), with a mean age of 26.16 ± 5.01 years (Table 2).

#### 3.1.2. Physiotherapy Interventions

All of the studies [17,21,22,23] but one [16] conducted follow-up for measuring their endpoints at various moments in time, always 7 days or more after the surgery. All of the studies presented an experimental group in which some physiotherapy intervention was applied. One study employed phototherapy (LED and low-intensity laser) [22], one study used electrotherapy (transcutaneous electrical nerve stimulation, TENS) [16], two studies used different approaches based on therapeutic exercise [17,21] and one study employed manual lymphatic drainage [23]. In the control groups, the same procedure was conducted as in the experimental group, but partially; for example, in one study [17], the control group did not perform a chewing exercise while the other group performed this exercise along with physiotherapy. Only in one study [16] did the control group undergo a different treatment than the intervention group. Table 3 describes the physiotherapy interventions performed in each of the studies.

#### 3.1.3. Variables of the Clinical Trials

- Mouth opening: measured as the maximum interincisal distance using a metal ruler or caliper [16,17].
- Neurosensory impairment: evaluated using five tests that assessed the patient’s ability to discriminate external sensory stimuli, using a visual analog scale (VAS) with five levels (one point, total absence of sensation; two points, almost no sensation; three points, reduced sensation; four points, almost normal sensation; five points: completely normal sensation). The five neurosensory tests were divided into three levels depending on their difficulty. The easiest level consisted of discriminating two points using a caliber and directional discrimination of the stimuli applied with a brush. The intermediate level consisted of recognizing the size of the Semmes–Weinstein monofilaments employed. The most difficult level consisted of thermal discrimination performed with ethyl chloride spray and discriminating nociceptive stimuli with a needle compared with a cotton swab [22].
- Myoelectric activity of the masticatory muscles: performed using an electromyography analysis, mainly in the masseter, temporal, sternocleidomastoid and anterior belly of the digastricus muscles [17,21].
- Pain: measured using the VAS, a 10 cm scale where one end represents the absence of pain and the other represents unbearable pain [23].
- Mouth strength: The measurement was performed using a GM10 occlusal force meter (Nagano Keiki Co.) [17].
- Facial edema: measured with a flexible plastic tape measure employing a procedure based on four separate lines: (1) mandibular angle–external corner of the eye; (2) mandibular angle–internal corner of the eye; (3) mandibular angle–mental protuberance; and (4) mental protuberance–external corner of the eye [23].

#### 3.1.4. Assessment of the Trials’ Methodological Quality

After assessing the studies’ methodological quality according to the PEDro scale, one RCT [22] showed good methodological quality, with a score of seven on the PEDro scale. One study achieved a score of six points [23], showing acceptable methodological quality, while the three remaining studies showed insufficient methodological quality, with scores of five points [16,17] and three points [21] on the PEDro scale (Table 1). The mean total score for methodological quality was 5.3 ± 1.37 points (range, 3–7).

The intervention of a third independent evaluator was needed to reach consensus in the evaluation of the methodological quality of one study [22]. The level of agreement between the evaluators according to the κ coefficient was high (κ = 0.82).

### 3.2. Risk of Bias

**Figure 2:**
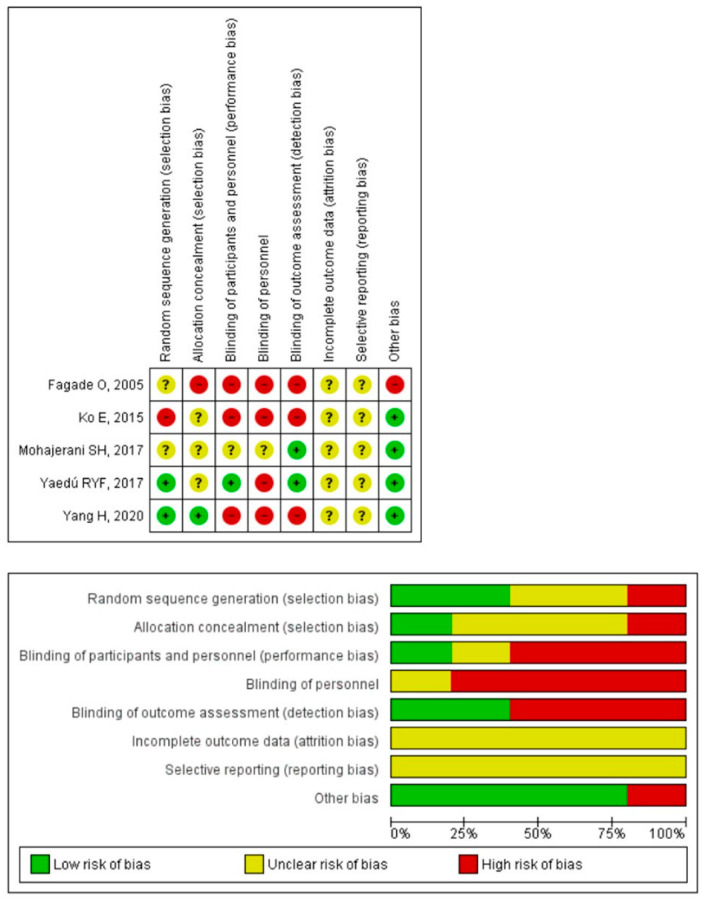
Plots of risk of bias of the included studies [16,17,21,22,23].

Only one of the studies performed double blinding (evaluator and patients) correctly [23], and another study performed a simple blind of the evaluator correctly [22]. None of the included studies properly indicated whether the results were obtained from the entire sample, whether there were losses during follow-up or whether there was an intent to treat in case of losses. Additionally, one of the studies showed another bias for finding statistically significant differences between the two groups before the intervention [16] (Figure 2).

### 3.3. Qualitative Analysis

In terms of the qualitative analysis of the results according to the level of evidence, we grouped only the studies that presented clinical and methodological homogeneity with each other.

#### 3.3.1. Range of Motion

There is limited evidence (one study [16], *n* = 20) showing that the application of TENS after orthognathic surgery, followed by an immobilization process, increases the ROM of the mouth opening, although in equal measure as in the control group, who took paracetamol.

There is limited evidence (one study [17], *n* = 22) showing that the application of physiotherapy combined with masticatory exercises improved the mouth opening, although in equal measure as in the control group, who did not perform the masticatory exercises.

#### 3.3.2. Neurosensory Impairment

There is moderate evidence (one study [22]; *n* = 20) showing that the combination of low-frequency laser with LED light reduces the potential complications of the inferior alveolar nerve after surgery compared with the control group, who were administered only LED light. Changes were recorded between the intervention and control groups in all of the subjective neurosensory assessment tests (VAS) and in the two objective neurosensory assessment tests (sensitivity when touching with a brush and discrimination of two points). These changes were maintained up to 2 months (discrimination of two points) and up to 6 months after the surgery (VAS and sensitivity when touching with a brush).

#### 3.3.3. Myoelectric Activity of the Masticatory Muscles

There is conflicting evidence (two studies [21], *n* = 63; [17], *n* = 22) on the results of myoelectric activity of the masticatory muscles after an intervention based on conventional physiotherapy and therapeutic exercise. The study by Ko et al. (2015) [21] observed that a program of therapeutic exercise and diet started 1 week after the surgery resulted in faster and greater rehabilitation of myoelectric activity in the masticatory muscles than in the control group, who only performed the diet. In contrast, the study by Yang et al. (2020) [17] observed that none of the two interventions produced changes in the myoelectric activity of the evaluated muscles.

#### 3.3.4. Pain

There is limited evidence (one study [23], *n* = 30) showing that manual lymphatic drainage does not produce significant changes in the perceived pain intensity evaluated with VAS.

#### 3.3.5. Bite Strength

There is limited evidence (one study [17], *n* = 22) showing that the application of physiotherapy combined with masticatory exercises improves bite strength, although in equal measure as in the control group, who did not perform the masticatory exercises.

#### 3.3.6. Facial Edema

There is limited evidence (one study [23], *n* = 30) showing that manual lymphatic drainage results in faster and greater resolution of facial edema after surgery when compared with placebo. However, there were no significant changes in the edema perceived by the patients measured with VAS.

## 4. Discussion

This is the first systematic review to evaluate the effects of physiotherapy interventions on postoperative ROM after orthognathic surgery, covering five studies and 155 participants. For the primary endpoint (ROM), there is limited evidence for the use of TENS and for the use of conventional physiotherapy and exercise for increasing ROM after surgery. For the secondary endpoints (myoelectric activity, pain, inflammation and bite strength), there is limited evidence for the use of manual lymphatic drainage for reducing postoperative inflammation, limited evidence for the use of conventional physiotherapy and exercise for increasing bite strength, moderate evidence for the use of LED and laser light in reducing sensory abnormalities of the inferior alveolar nerve and limited evidence for the use of exercise in increasing myoelectric activity after orthognathic surgery.

### 4.1. Range of Motion

Two studies used ROM as the endpoint [16,17], but used different interventions. For the study by Fagade et al. (2005), the results from applying TENS agree with those found in other pain conditions, such as cervical pain, in which TENS was observed to improve ROM but was not superior to other interventions [26]. Rakel et al. (2014) concluded that TENS is no better than placebo in managing pain and ROM restriction after total knee arthroplasty, also indicating that the patients with better results after TENS were those with lower levels of anxiety or catastrophism [27]. The fact that orthognathic surgery patients have greater social anxiety levels than the rest of the population [28] could explain why Fagade et al. (2005) [16] observed no ROM improvement in the patients who underwent TENS. Additionally, the use of paracetamol is recommended for managing postsurgical pain [29], and pain has been considered a factor related to restricted ROM of the jaw [30], which could be related to the lack of difference between the TENS group and the paracetamol group.

In the study by Yang et al. (2020) [17], the lack of differences between the groups could be due to the fact that the control group also performed the mobility exercises, which are recommended for treating restricted ROM in other maxillofacial surgeries, such as temporomandibular joint surgery [31]. Additionally, the dose of isometric exercises might have increased masticatory muscle fatigue in the intervention group, which could be related to the reduced mandibular function [32], both in restricted mobility and bite strength.

### 4.2. Neurosensory Impairment

Only Mohajerani et al. (2017) assessed the effect of laser therapy and LED on this outcome after orthognathic surgery [22]. The authors’ results agree with the results from other studies: laser therapy improved the sensitivity of the inferior alveolar nerve [33]. These results might be due to the effect of laser therapy on the immune response and the regeneration of peripheral nerve axons, including those of the inferior alveolar nerve, observed in animal models [34].

### 4.3. Myoelectric Activity of the Masticatory Muscles

There is conflicting evidence for the use of conventional physiotherapy and exercise in the rehabilitation of myoelectric activity of the masticatory muscles. The differences between the two studies that analyzed this endpoint [17,21] were probably due to the fact that the time interval between the surgery and the start of the intervention in the two studies differed greatly (Ko et al. (2015) [21] started the physiotherapy 8 days after the surgery, while Yang et al. (2020) [17] stated it 3 weeks after the surgery). Although the early start of physiotherapy has not been studied after orthognathic surgery, it has been studied in other types of surgery in the maxillofacial region, such as temporomandibular joint surgery. De Meurechy et al. (2019) [31] conducted a systematic review and concluded that postoperative physiotherapy benefited patients’ rehabilitation, starting between 24 h and 1 week after the surgery. In their study, Abboud et al. (2018) [35] concluded that the immediate start of exercises produced better effects than the gradual start of exercises.

### 4.4. Pain and Facial Edema

Lastly, another study included in this systematic review assessed the changes produced by manual lymphatic drainage in edema and pain [23]. The change in the objective measures of facial edema was similar to those observed by Van de Velde et al. (2020) [36]. The group that underwent treatment with lymphatic drainage had a faster reduction of the edema (although in this case the difference was not statistically significant). However, the lack of changes in the patients’ perceived pain could be due to the intervention of the control group that underwent a manual lymphatic drainage placebo, given that it has been shown that touch produces the inhibition of cortical and subcortical nociceptors [37].

The main limitations of this systematic review were the methodological quality of the RCTs, given that, after analyzing them with the PEDro scale, only two studies [22,23] presented an acceptable methodological quality, with scores of seven and six points, respectively [19]. Additionally, all the studies had a moderate to high risk of bias. Furthermore, the limited number of studies included in the systematic review should be considered a limitation. Lastly, we rejected one study due to being written in Korean [38], which should be considered a limitation as well.

Moreover, it was impossible to perform a meta-analysis due to the low number of high-quality studies and the considerable heterogeneity of the measurement variables employed in the various study endpoints.

### 4.5. Clinical Implications

From a critical standpoint, we currently have insufficient scientific evidence to support the clinical use of physiotherapy for patients who have undergone orthognathic surgery. This systematic review suggests that physiotherapy and a number of its techniques might be useful in these patients’ rehabilitation process. Nevertheless, the clinical reality highlights the limitations and obstacles for ensuring diligent and high-quality access to physiotherapy units. It would be interesting to have systems for directly accessing specialized physiotherapy units by all medical specialties, as well as for clinics and hospitals having this treatment in their portfolio of patient services. Although the evidence is limited in this area of intervention, early physiotherapy has already widely shown its efficacy in rehabilitating surgical patients.

## 5. Conclusions

The effects of the physiotherapy interventions studied in this systematic review on the variables of range of motion, myoelectric activity, pain, inflammation and masticatory muscle strength are limited. There is limited evidence for the use of TENS, conventional physiotherapy and exercise for increasing mouth opening ROM. Moreover, there is conflicting evidence on the effects of a program of mobility and isometric exercises on myoelectric activity. The only intervention that has shown a moderate level of evidence is the intervention based on laser therapy and LED in managing sensory abnormalities of the inferior alveolar nerve.

## Figures and Tables

**Table 1:** Methodological score of randomized clinical trials using the Physiotherapy Evidence Database scale (PEDro).

PEDro Scale Items	1	2	3	4	5	6	7	8	9	10	11	Total
Fagade et al., 2005 [16]	1	1	0	0	0	0	0	1	1	1	1	5
Ko et al., 2015 [21]	1	0	0	1	0	0	0	0	0	1	1	3
Mohajerani et al., 2017 [22]	1	1	0	1	0	0	1	1	1	1	1	7
Yang et al., 2020 [17]	1	1	1	1	0	0	0	0	0	1	1	5
Yaedú et al., 2017 [23]	1	1	0	1	1	0	1	0	0	1	1	6

Items. 1: Eligibility criteria were specified; 2: subjects were randomly allocated to groups; 3: allocation was concealed; 4: the groups were similar at baseline regarding the most important prognostic indicators; 5: there was blinding of all subjects; 6: there was blinding of all therapists who administered the therapy; 7: there was blinding of all assessors who measured at least one key outcome; 8: measures of at least one key outcome were obtained from more than 85% of the subjects initially allocated to groups; 9: all subjects for whom outcome measures were available received the treatment or control condition as allocated or, where this was not the case, data for at least one key outcome were analyzed by “intention to treat”; 10: the results of between-groups statistical comparison are reported for at least one key outcome; 11: the study provides both point measures and measures of variability for at least one key outcome.

**Table 2:** Participant characteristics of the included trials and effects of interventions.

	Demographic Data	IG	CG	Key Outcomes	Assessment	Conclusions
Fagade et al., 2005 [16] PEDro: 5	G1 (*n* = 10), M: 6, F: 4, 34.5 ± 10.37 years on average G2 (*n* = 10), M: 4, F: 6, 36.2 ± 14.27 years on average	G1: TENS G2: paracetamol	There was no control group	MMO	A caliper was used to measure interincisal distance	G1: SSI in MMO Paracetamol: SSI in MMO G1 vs. G2: without statistically significant differences.
Ko et al., 2015 [21] PEDro: 3	IG (*n* = 31), M: 9, F: 22, 24 ± 3.6 years on average CG (*n* = 32), M: 8, F: 24, 25.3 ± 4.8 years on average	Diet and PT program	Diet	Myoelectric activity of masticatory muscles	Surface EMG (Zebris EMG 4, Zebris gmbH, Isny im Allgäu, Germany) and software for analyzing myoelectric signal (WinJaw 10.5 Zebris GmbH, Isny im Allgäu, Germany)	IG vs. CG: SSI in favor of IG in myoelectrical activity recovery of masticatory muscles
Mohajerani et al., 2017 [22] PEDro: 7	IG (*n* = 10), M: 5, F: 5, 24.1 ± 4.6 years on average CG (*n* = 10), M: 3, F: 7, 22.8 ± 3.6 years on average	LIL + LED	LED	Neurosensory Recovery	It was assessed by using a clinical neurosensory test including brush stroke allodynia, 2-point discrimination, contact detection, pinprick nociception and thermal discrimination. In addition, neurosensory recovery was subjectively measured using a VAS scale	IG: SSI in VAS score, brush stroke allodynia and 2-point discrimination IG vs. CG: SSI in favor of IG in neurosensory recovery of subjects. SSI in favor of IG in VAS score, brush stroke allodynia in 6-month follow up and in 2-point discrimination in the 2-month follow up.
Yang et al., 2020 [17] PEDro: 5	IG (*n* = 12), M: 7, F:5, 22.3 ± 4.3 years on average CG (*n* = 10), M: 5, F:5, 21.9 ± 2.9 years on average	Standard PT + therapeutic exercise program.	Standard PT	Bite force, MMO, myoelectric activity	Bite force assessed with a specific device (Occlusal force-meter GM10, Nagano keiki Co., Ltd., Tokyo, Japan) MMO assessed with a ruler (interincisal distance) Myoelectric activity assessed with an electromyograph (BioEMG II Bioresearch Assoc., Milwaukee, WI, USA)	IG: SSI in bite force and MMO CG: SSI in bite force and MMO IG vs. CG: no differences
Yaedú et al., 2017 [23] PEDro: 6	IG (*n* = 15), M: 12, F: 3, 25.67 ± 6.41 years on average CG (*n* = 15), M: 12, F: 3, 24.87 ± 3.18 years on average	Manual lymphatic drainage, cryotherapy, medication	Placebo lymphatic drainage, cryotherapy, medication	Edema and patient perception of edema and pain intensity	Edema was assessed with tape and photographs. Patient perception of edema and pain intensity were assessed with a VAS	IG: SSI in edema regression IG vs. CG: no differences

G1: intervention group 1. G2: intervention group 2. IG: intervention group. CG: control group. PT: physiotherapy. M: male. F: female. MMO: maximum mouth opening. SSI: statistically significant improvement. EMG: electromyography. LIL: low-intensity laser. VAS: visual analogue scale. LED: light-emitting diode. TENS: transcutaneous electrical nerve stimulation.

**Table 3:** Characteristics of the interventions.

RCT	Intervention	Description
Fagade et al., 2005 [16]	TENS	TENS (100 µs width pulse, 50 Hz frequency) was applied using circular electrodes of 3 cm. The positive electrode was placed in masseter muscle and the negative in zygomatic bone. TENS intensity was adjusted based on the tolerance level of each patient, but without visible muscle contraction. The intervention lasted 30 min. Paracetamol was administrated to the control group.
Ko et al., 2015 [21]	Therapeutic Exercise	Therapeutic exercise intervention started on the 8th postsurgical day. During the first three weeks, intervention protocol included active mobility exercises (jaw opening 6 times of 30 s; lateralization 10 times of 5 s; protrusion 10 times of 5 s). After each session, patients were allowed to self-massage masticatory muscles. From the 5th post-surgical week, isometric contraction exercises were included (3 times of 10 s). Control group did not receive exercise intervention.
Mohajerani et al., 2017 [22]	LIL + LED	LIL of 810 nm, energy intensity of 5 J/cm^2^ LED of 632 nm, energy intensity of 2 J/cm^2^ The intervention was applied in four different locations (mandibular foramen, mandibular body, lips and chin) for 90 s each. The intervention was applied during the 1st, 2nd, 3rd, 7th, 14th, 28th days after the surgery. The control group received only LED.
Yang et al., 2020 [17]	Therapeutic Exercise	Therapeutic exercise intervention started on the third postsurgical week. Patients were instructed to use their first and second finger to self-assess opening jaw movement and to do active lateralization movements during 5 to 10 min. In addition, isometric contraction exercises were included using a specific device (NoSick, Hi-Feel World Co., Ltd., Seoul, Korea). Patients were instructed to bite the device 200 times, 3 times a day, with best occlusion possible. Control group did do not the isometric contraction exercises
Yaedú et al., 2017 [23]	MLD	MLD was applied over 5 consecutive days, always in the morning, starting on the second postsurgical day. The MLD technique was carried out in a relaxed environment, with the patient laying in supine position, head raised 30° and the physiotherapist conducted the Leduc method with a slight pressure (30–40 mmHg). Control group received placebo MLD, which consisted of superficial lymphatic drainage.

RCT: randomized clinical trial. TENS: transcutaneous electrical nerve stimulation. LIL: low-intensity laser. LED: light-emitting diode. MLD: manual lymphatic drainage.

## Data Availability

No new data were created or analyzed in this study. Data sharing is not applicable to this article.
